# Investigations on the Usefulness of CEACAMs as Potential Imaging Targets for Molecular Imaging Purposes

**DOI:** 10.1371/journal.pone.0028030

**Published:** 2011-12-05

**Authors:** Markus Heine, Peter Nollau, Christoph Masslo, Peter Nielsen, Barbara Freund, Oliver T. Bruns, Rudolph Reimer, Heinrich Hohenberg, Kersten Peldschus, Harald Ittrich, Udo Schumacher

**Affiliations:** 1 Institute of Anatomy and Experimental Morphology, University Hospital Hamburg-Eppendorf, Hamburg, Germany; 2 Department of Clinical Chemistry, Center of Clinical Pathology, University Hospital Hamburg-Eppendorf, Hamburg, Germany; 3 Institute of Biochemistry and Molecular Biology II: Molecular Cell Biology, University Hospital Hamburg-Eppendorf, Hamburg, Germany; 4 Heinrich-Pette-Institute for Experimental Virology and Immunology at the University of Hamburg, Hamburg, Germany; 5 Department of Diagnostic and Interventional Radiology, University Hospital Hamburg-Eppendorf, Hamburg, Germany; Genentech, United States of America

## Abstract

Members of the carcinoembryonic antigen cell adhesion molecules (CEACAMs) family are the prototype of tumour markers. Classically they are used as serum markers, however, CEACAMs could serve as targets for molecular imaging as well.

In order to test the anti CEACAM monoclonal antibody T84.1 for imaging purposes, CEACAM expression was analysed using this antibody. Twelve human cancer cell lines from different entities were screened for their CEACAM expression using qPCR, Western Blot and FACS analysis. In addition, CEACAM expression was analyzed in primary tumour xenografts of these cells. Nine of 12 tumour cell lines expressed CEACAM mRNA and protein when grown *in vitro*. Pancreatic and colon cancer cell lines showed the highest expression levels with good correlation of mRNA and protein level. However, when grown *in vivo*, the CEACAM expression was generally downregulated except for the melanoma cell lines. As the CEACAM expression showed pronounced expression in FemX-1 primary tumours, this model system was used for further experiments. As the accessibility of the antibody after i.v. application is critical for its use in molecular imaging, the binding of the T84.1 monoclonal antibody was assessed after i.v. injection into SCID mice harbouring a FemX-1 primary tumour. When applied i.v., the CEACAM specific T84.1 antibody bound to tumour cells in the vicinity of blood vessels. This binding pattern was particularly pronounced in the periphery of the tumour xenograft, however, some antibody binding was also observed in the central areas of the tumour around blood vessels. Still, a general penetration of the tumour by i.v. application of the anti CEACAM antibody could not be achieved despite homogenous CEACAM expression of all melanoma cells when analysed in tissue sections. This lack of penetration is probably due to the increased interstitial fluid pressure in tumours caused by the absence of functional lymphatic vessels.

## Introduction

Members of the carcinoembryonic antigen (CEACAM) family are transmembrane glycoproteins belonging to the immunoglobulin superfamily, which are involved in a variety of biological processes [1], [2]. These include regulation of cell growth, differentiation, immune response, cellular recognition and cell adhesion [3], [4], [5], [6]. In addition to their normal function, expression of several members of the CEACAM family was found to be upregulated in colorectal and lung cancer as well as in melanoma [7], [8], [9]. Due to their up-regulation in these entities, members of the CEACAM family have served as valuable clinical markers both in tissue sections and patients' sera [10], [11]. In particular, the classical serum marker CEACAM5 (CEA) is highly expressed in cancers including colorectal, gastric, pancreatic, and small cell lung cancer [12], [13], [14]. Because of their high expression level in colon cancer, serum CEA levels are routinely used to monitor the recurrence of colonic adenocarcinoma after surgery and some of the antibodies have been used in patient studies [15], [16], [17].

However, marker analysis of serum samples does not disclose the site of CEA production and therefore the site of the (primary) tumour remains unresolved by serum analysis. To localise tumours, endoscopic as well as non-invasive imaging techniques like MRI are used, which, however, lack information about the specific proteins secreted by the tumour including CEA. To obtain information about the specific molecular composition of tumours, MRI techniques have to be combined with antibody based technologies resulting in molecular imaging techniques [18].

To discover the capabilities and limitations of molecular imaging we developed murine xenograft models for *in vivo* detection of CEACAMs. CEACAMs were chosen for targeting as they are often highly expressed in a variety of malignancies (see above). In order to broaden the specificity of our molecular probe, we used the T84.1 monoclonal antibody which is capable of recognising several members of the CEACAM family including CEACAM 1, 5 and 6 [19].

This contribution describes the expression of T84.1 immunoreactivity in 12 different human cancer cell lines for CEACAM expression *in vitro* and when grown in immunodeficient mice *in vivo* as primary tumour in order to establish a xenograft model for CEACAM detection. With one of these models we additionally investigated the accessibility of CEACAMs to antibodies in the primary tumour after i. v. application of the anti pan-CEACAM antibody T84.1.

## Materials and Methods

### Cell lines

The human prostate cancer cell lines LNCAP [20] and PC3 [21] (both established from metastatic adenocarcinomas) were obtained from the German Collection of Microorganisms and Cell Culture (DSMZ, Germany). The human breast cancer cell lines T47D [22] and MCF7 [22] (both established from pleural effusions) were obtained from European Cell Culture Collection (Porton Down, Wiltshire, UK). The human melanoma cell lines MEWO [23] and FemX-1 [24] (both established from metastatic melanoma lymph nodes) were kindly provided by the Klinik für Dermatologie, Universitätsklinikum Hamburg-Eppendorf, Germany. The human colon cancer cell line HT29 [25] (established from a primary carcinoma of the colon) was obtained from Cell Lines Service (Germany). The human colon cancer cell lines Caco2 and SW480 [22] (both established from primary adenocarcinomas of the colon) were obtained from European Cell Culture Collection (Porton Down, Wiltshire, UK). The human small cell lung cancer cell line OH-1 [26] (established from pleural effusion) was kindly provided by Prof. Uwe Zangemeister-Wittke, University of Berne, Department of Pharmacology. The human pancreatic cancer cell line 5061 [27] (established from a advanced pancreatic adenocarcinoma) was kindly provided by the Klinik und Poliklinik für Allgemein-, Viszeral- und Thoraxchirurgie, Universitätsklinikum Hamburg-Eppendorf, Germany. The human prancreatic cell line 5072 (established from a advanced pancreatic adenocarcinoma from a 71-year-old Caucasian woman) was kindly provided by the Klinik und Poliklinik für Allgemein-, Viszeral- und Thoraxchirurgie, Universitätsklinikum Hamburg-Eppendorf, Germany. Histopathological examination of the surgical specimen confirmed a low-differentiated adenocarcinoma of the pancreas, which was staged pT3, pN2, G3, M0, R0. Written informed consent of the patient for the removal of tissue samples for investigational purposes was obtained prior to surgery. The study was approved by the ethical committee of the Medical Council of Hamburg (Ärztekammer), Germany.

The cell lines LNCAP, PC3, T47D, MCF7, MEWO, FemX-1, HT29, Caco2, SW480, OH-1 were cultured in vitro under standard cell culture conditions (37°C, 100% relative humidity, 5% CO_2_) in RPMI medium (Gibco/Life Technologies, Paisley, Scotland) supplemented with 10% heat inactivated fetal bovine serum (FBS, Gibco), 2 mM L-glutamine (Gibco), 100 U/ml penicillin and 100 µg/ml streptomycin (Gibco). The cell lines 5061 and 5072 were cultured in complete (TUM) RPMI 1640 medium with Glutamax (Invitrogen, NY, USA) supplemented with 10% of fetal calf serum (FCS), 200 IU/ml of penicillin-streptomycin, 0.1 mg/ml gentamycin (Biochrom AG, Berlin, Germany), 50 nmol/ml of human transferrin (Sigma-Aldrich, Steinheim, Germany), 0.01 µg/ml of bovine insulin (Sigma-Aldrich, Steinheim, Germany), 0.01 µg/ml of recombinant human epidermal growth factor (Pepro Tech, London, UK), and 0.01 µg/ml of human basic fibroblast growth factor (Pepro Tech, London, UK). Before reaching confluence, cells were routinely harvested for passaging using 0.05% trypsin-0.02% EDTA (Gibco).

### Real-time PCR

To quantify CEACAM mRNA amount in relation to GAPDH mRNA amount of the human tumour cell lines, real-time PCR was carried out. In brief, total RNA from tumour cells was isolated using RNeasy Mini Kit (Qiagen, Hilden, Germany) according to the manufacturer's instructions. The RNA was eluted in 50 µl RNase free water. The RNA-concentration was measured and the quality was checked on a NanoDrop® ND-1000 Spectrophotometer (Peqlab, Erlangen, Germany). The cDNA synthesis was performed in a Biometra thermal cycler (Biometra, Göttingen, Germany) in a total volume of 20 µl for each sample and followed the manufacturer's instruction for the First Strand Transcriptor cDNA Synthesis Kit (Roche Diagnostics, Mannheim, Germany). Two parallel cDNA approaches were used for reverse transcription separately, with anchored-oligo(dT)18 and random hexamer primer. Two µg of total RNA were used for each cDNA approach and were pooled afterwards. Real-time polymerase chain reaction was performed in a 96 well format with the LightCycler® 480 System (Roche Diagnostics GmbH, Mannheim, Germany). For the real-time PCR, the LightCycler Fast Start DNA MasterPLUS SYBRGreen I Kit (Roche Diagnostics GmbH, Mannheim, Germany) was used. Two µl of cDNA was used as a template for the PCR reaction and incubated in a total reaction volume of 10 µl, containing 1×SYBR Green I Master mix including Taq DNA polymerase, Taq PCR buffer, a dNTP mixture and 1 mmol/l MgCl_2_, 10 pmol specific CEACAM or GAPDH primers. Forward CEACAM primer (TGT GAA TGA AGA AGC AAC), reverse CEACAM primer (CAG CCT GGG ACT GAC CGG), forward GAPDH primer (AAA TTG AGC CCG CAG CCT CCC), and reverse GAPDH primer (CCA GGC GCC CAA TAC GAC CAA AT) were synthesized by MWG-BIOTECH AG (Ebersberg, Germany). The PCR conditions were initially 5 min 95°C, followed by 40 cycles of 10 s 95°C, 10 s 62°C (CEACAM) or 72°C (GAPDH) and 20 s 72°C, respectively. The primer pair detects in parallel CEACAM 1, 5 and 6.

### Protein extraction and Western blotting

Total protein extracts from cell lines were obtained by lysing the cells in cold radioimmunoprecipitation assay (RIPA) buffer (50 mM Tris, 2 mM EDTA, 1% NP-40, 0.1% SDS, and 150 mM NaCl) in the presence of protein inhibitor cocktail set I (Calbiochem, La Jolla, USA). After centrifugation to remove cell debris, protein concentrations of the supernatants were measured using the BCA method [28]. 40 µg protein per lane were resuspended in loading buffer (0.5 M Tris-HCl, pH 6.8, glycerol, 10% SDS, 0.5% bromphenol blue, mercaptoethanol) and then subjected to sodium dodecyl sulfate–polyacrylamide gel electrophoresis (8% gels). Proteins were subsequently blotted onto a nitrocellulose membrane (Hybond®-ECL®, Amersham Biosciences, Freiburg, Germany) following conventional protocols. Finally, blots were blocked in 4% bovine serum albumin/Tris-buffered saline–0.1% Tween 20 (TBS-T) for 30 min at room temperature. Membranes were incubated with primary antibody T84.1 (1 µg/ml) and anti-beta-Actin (0.5 µg/ml, Abcam, Cambridge, UK) over night at 4°C, washed with TBS-T and incubated with a 1∶200 diluted polyclonal goat-anti-mouse antibody (DakoCytomation, Carpinteria, USA) conjugated with horseradish peroxidase for 60 min at room temperature. The bound immune complexes were visualised using ECL Western Blotting Substrate (Pierce, Rockford, USA) and the ChemiDoc XRS System (Bio-Rad, Munic, Germany).

### Flow Cytometry

Cultured cells were detached with Cell Dissociation Buffer (GIBCO™, Carlsbad, US) and incubated on ice for 30 min with T84.1 primary antibody at a concentration of 1 µg/ml. The corresponding murine isotype control was IgG1 (DakoCytomation, Carpinteria, USA). The cells were washed and the primary antibody was detected with allophycocyanin-conjugated goat-anti-mouse antibody (Becton Dickinson Biosciences, Heidelberg, Germany). Flow cytometry was performed using a FACS CALIBUR flow cytometer (Becton Dickinson, Heidelberg, Germany). Data were analyzed using Win MDI 2.9 software.

### Immunochemistry

For immunocytochemistry, cells cultured in eight-well chamber slides (Becton Dickinson, Heidelberg, Germany) were washed with PBS, fixed in ice-cold acetone for 2 min, air dried, and blocked with 10% rabbit sera in blocking reagent (TBS). Slides then were incubated with T84.1 or IgG1 control antibody at a dilution of 0,6 µg/ml over night (4°C) followed by a biotinylated rabbit anti mouse antibody (DakoCytomation) at a dilution of 1∶200 for 30 min. After careful washes in TBS, an incubation with an avidin-alkaline phosphatase complex (ABC kit, Vectastain, Vector, Burlingame, CA) for 30 min followed and thereafter, additional washes in TBS were performed. Alkaline phosphatase activity was visualised using Naphthol-AS-bisphosphate as a substrate and New Fuchsin was used for simultaneous coupling. Slides were counterstained with Mayer's hemalum diluted 1∶1 in distilled water for ten seconds, blued under running tap water and mounted with Aquatex® (Merck KGaA, Darmstadt, Germany). The intensity (plus signs) and extent (percentage) of the positive areas of five histological sections were determined by visual inspection of three independent observers. The plus sign indicates the intensity of the staining. It ranges from “+++” to “−”. The percentage indicates how many cells are labelled.

### Immunohistochemistry of tumour cell lines xenotransplanted in SCID mice

Sections of all primary tumors were drawn from our in house tumor bank. They had all been fixed with 4% formalin and embedded in paraffin wax according to standard procedures. For immunohistochemistry, 5 µm thick sections were cut, dewaxed and microwaved in 10 mM citrate buffer (pH 6.0) at 500 W five times for 2 min and then cooled for 20 min. After washing, non-specific binding was blocked by incubating the sections in 10% normal rabbit serum (DAKO, Hamburg, Germany) for 30 min at room temperature. The following steps were as described above.

### Ethics Statement

The methodology for carrying out the experiment was consistent with the UKCCCR guidlines for the welfare of animals in cancer research [29]. The experiment was supervised by the institutional animal welfare officer and approved by the local licensing authority (Behörde für Soziales, Familie, Gesundheit und Verbraucherschutz; Amt für Gesundheit und Verbraucherschutz, Hamburg, Germany, project no. 92/09).

### In vivo experiments

To investigate *in vivo* CEACAM binding sites, specific pathogen-free BALB/c SCID (scid/scid) mice were used. The mice were 8–16 weeks old and weighed 20–30 g at the beginning of our experiments. They were housed in filter top cages and provided with sterile water and food ad libitum. For injection, FemX-1 melanoma cells were harvested by trypsination and viable cells (5×10^6^) were suspended in 1 ml of cell culture medium. An aliquot of 200 µl of this suspension was injected subcutaneously between the scapulae of each SCID mouse. Twelve mice bearing melanomas were included in the experiment when the tumour had reached maximal growth or started to ulcerate.

Iodination of antibodies was done by Iodobeads (Pierce, Rockford, USA). Specifically, 5 µl sodium [^125^I]Iodine (3.7 MBq/µl, Amersham, UK) was added to 1 mg T84.1 or control IgG1 in 1 ml PBS in the presence of one Iodobead and the reaction was left for 15 min. Free [^125^I]Iodine was removed by exclusion chromatography through a PD-10 column (GE Healthcare, Buckinghamshire, UK). The protein concentration was determined by the BCA method and the specific activity of the [^125^I]-T84.1 and [^125^I]-IgG1 per µg protein was measured by gamma counting (Perkin Elmer, Waltham, USA).

Ten µg of [^125^I]-labelled antibodies were injected via the tail vein into FemX-1 melanoma bearing mice. Six mice received [^125^I]-IgG1 and six mice [^125^I]-T84.1 antibody. After 0.5, 2.5, 6, 17.5, and 22.5 hours blood samples were taken by tail vein to measure antibody blood half life. Afterwards mice were sacrificed, intracardially perfused with NaCl and tumour and organs were removed and weighted. Radioactivity of organs, tumours and blood were determined in the gamma counter. Statistical analyses (Two-way ANOVA) were performed using GraphPad Prism 5 software (GraphPad software, USA).

Evans Blue was injected i.v. at a dose of 25 µl (50 mg/ml PBS) per FemX-1 s.c. bearing mouse 4 hours before scarification. Anaesthetised mice were intracardially perfused with 1% BSA in NaCl to remove free dye and afterwards fixed with 4% PFA. Tumours were removed and embedded in 5% agarose gel, cut in 200 µm thick slices with a vibratome (VT1000E, Zeiss, Wetzlar, Germany) and scanned with 2400 dpi on a scanner (Epson, Long Beach, USA).

## Results

### CEACAM expression in vitro

**Figure 1:**
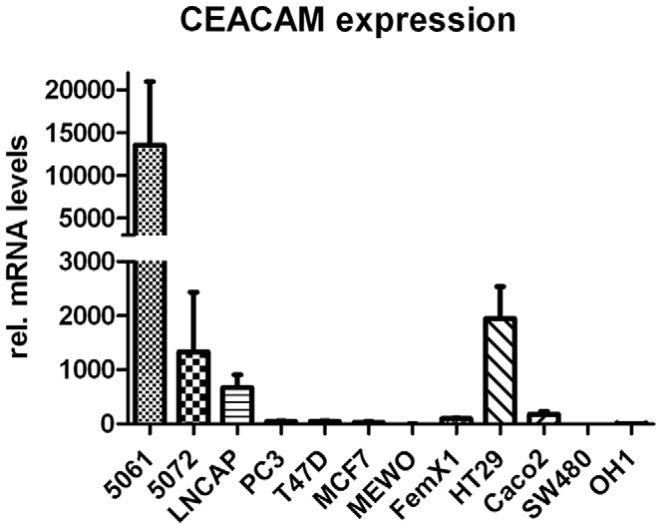
CEACAM mRNA expression of human malignant cells. The mRNA of the malignant cell lines 5061, 5072 (pancreas), PC3, LNCAP (prostate), FemX-1, MEWO (melanoma), MCF7, T47D (breast), HT29 (colon) and OH-1 (small cell lung cancer) were relatively quantified by real time PCR, using GAPDH for normalization. Cell lines 5061, 5072, LNCAP and HT29 showed high expression levels of CEACAM mRNA.

The CEACAM expression levels of twelve human cancer cell lines derived from different tumour entities are summarized in figure 1 and table 1.

**Table 1:** Summary of *in vitro* and *in* vivo CEACAM expression of all cell lines by different methods.

	cell culture				xenografted tumor
	QPCR	WB	FACS positive	immunocytochemistry	immunohistochemistry
5061	+++	+	99%	+++ (95%)	++/+++ (45%)
5072	++	+	77%	+++ (90%)	− (0%)
LNCAP	++	+	93%	++ (100%)	++/+++ (60%)
PC3	+	+	23%	++ (10%)	(+) (20%)
T47D	+	(+)	5%	+ (1%)	+/++ (20%)
MCF7	+	+	23%	+++ (5%)	+/++ (20%)
MEWO	(+)	+	74%	+++ (85%)	++ (60%)
FemX-1	+	+	95%	+++ (100%)	++/+++ (90%)
HT29	++	+	86%	+++ (90%)	++ (45%)
Caco2	+	+	16%	+++ (90%)	++ (20%)
SW480	−	+	31%	+ (5%)	(+) (30%)
OH-1	(+)	+	80%	++ (80%)	(+) (100%)

Note that the highest CEACAM expression *in vitro* and *in vivo* showed the melanoma cell line FemX-1. The intensity (plus signs) and extent (percent) of the positive areas of 5 histological sections were determined by visual inspection of 3 independent observers.

The highest expression of CEACAM mRNA was detected in the pancreatic tumour cell lines 5061 and 5072, the colon cancer cell lines HT29 and Caco2, and the prostate cancer cell line LNCAP. Low levels could be found in the breast cancer cell lines T47D and MCF7 and in the melanoma cell line FemX-1. Almost no CEACAM expression could be detected in the lung cancer cell line OH-1, the melanoma cell line MEWO, and the colon cancer line SW480.

**Figure 2:**
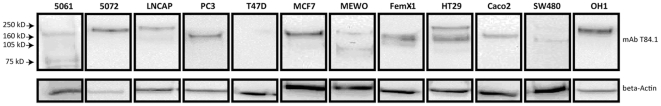
Western blot analysis of the CEACAM protein expression pattern. CEACAM family members as detected by pan CEACAM specific antibody T84.1 were present in all cancer cell lines except T47D. Beta-actin was used as a loading control.

Presence of the CEACAM proteins was analyzed by Western Blot of cell lysats (figure 2, table 1). All cell lines showed distinct protein bands except for the cell line T47D, which showed only a very faint band. Several bands were detected in the blots, presumably because of the different glycosylation isoforms that exists of the CEACAM proteins. No obvious correlation between mRNA and protein levels was observed.

**Figure 3:**
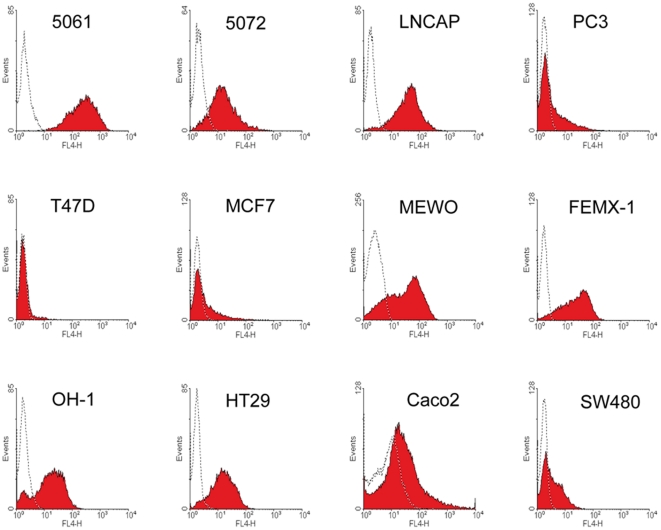
CEACAM cell surface presence of tumour cells. CEACAM could positively be detected by FACS-analysis with mAb T84.1 by all cancer cell lines, except of T47D, MCF7 and PC3. Isotype controls are shown as dotted lines.

The amount of membrane bound CEACAM proteins were furthermore determined by FACS analysis using mAb T84.1 (figure 3). As summarized in table 1, almost all cell lines showed the same staining pattern, as seen in the immunocytochemistry.

**Figure 4:**
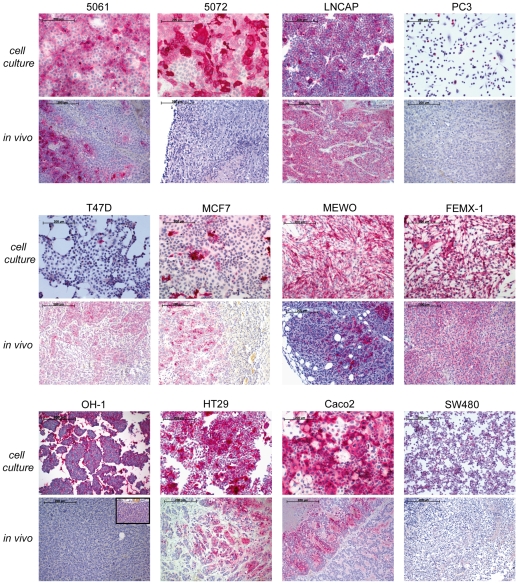
CEACAM protein expression pattern in vivo and in vitro. Cell lines LNCAP and FemX-1 showed *in vitro* and *in vivo* strong binding of mAb T84.1 by all cells. In contrast, cell lines 5061, MEWO, OH-1 (insert with higher magnification) HT29, Caco2, and particularly 5072 showed strong mAb T84.1 *in vitro* binding, but little or no binding *in vivo*. (red = T84.1 binding).

To visualize the CEACAM expression pattern under cell culture conditions we stained the cells, grown on chamber slides, with the panCEACAM T84.1. As shown in figure 4, the pancreatic (5061, 5072), colon (Caco2, HT29, SW480), and melanoma (FemX-1, MEWO) cancer cell lines clearly bound mAb T84.1. Lower or no binding could be detected by prostate (LNCAP, PC3) and breast (MCF7, T47D) cancer cell lines.

### CEACAM expression in vivo

Only FemX-1 and LNCAP primary xenograft tumours showed strong mAb T84.1 binding by more than 50% of all cells (figure 4 and table 1), while all other primary tumours did not show substantial T84.1 binding.

### CEACAM binding detection in vivo

**Figure 5:**
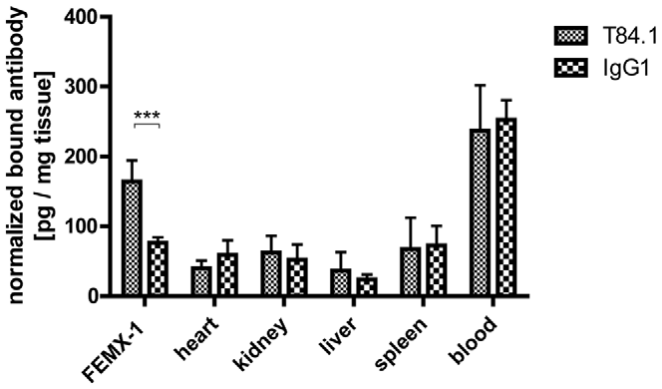
CEACAM in vivo binding. [^125^I]-Labelled T84.1 and nonspecific [^125^I]-labelled IgG1 antibody were used for CEACAM *in vivo* binding in FemX-1 melanoma bearing SCID mice. There is a significant difference between specific and control antibody by FemX-1 melanoma, but not in other organs including blood (Two-way ANOVA, P<0.001, n = 6).

**Figure 6:**
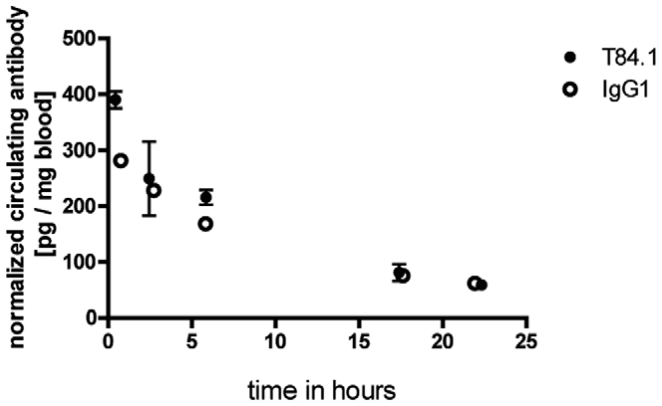
Blood half life of mAbs T84.1 and IgG1. The blood half life of [^125^I]-labelled T84.1 and nonspecific [^125^I]-labelled IgG1 antibodies were determined in FemX-1 tumour bearing SCID mice. The blood half life of specific and control antibody were identical (T84.1 = 10.3 h (n = 4), IgG1 = 10.4 h (n = 4)).

To analyse whether the CEACAM binding sites detected in tissue sections were also accessible *in vivo*, [^125^I]-labelled T84.1 mAb and non-specific [^125^I]-labelled control IgG were injected into the tail vein of FemX-1 melanoma bearing mice. A significant two-fold higher enrichment of the [^125^I]-T84.1 mAb in the FemX-1 melanoma was detected compared to the [^125^I]-IgG1 control (figure 5) (Two-way ANOVA, P<0.001). There was no significant difference concerning blood half life or tissue distribution between specific [^125^I]-T84.1 mAB and control [^125^I]-IgG (figure 6).

**Figure 7:**
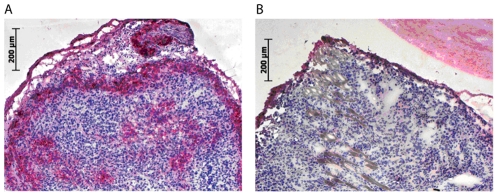
CEACAM in vivo detection of FemX-1 tumour. A: T84.1-antibody binds to FemX-1 tumour cells in vivo after i.v. injection of T84.1- (50 µg) in tumour bearing mice, as visualized in cryostat sections with subsequent immunostaining against T84.1-antibody (red = T84.1 positive cells) in sections of the primary tumour. B: Controls using IgG1 ab (40 µg) proved the high specificity of T84.1 binding to tumour cells in vivo. The specific antibody T84.1 binding was detected at the entire margins of the tumour, which is well supplied with blood vessels. In contrast, T84.1 binding was only observed around blood vessels in the central area of the tumour.

In a parallel experiment the distribution of the injected mAbs 24 hours after application were analysed in histological sections regarding tissue distribution of the antibody (figure 7). No detectable levels of antibody could be found in control animals injected with control IgG1 mAb. In contrast, T84.1 binding could be detected at the margin of the tumour and in the central area of the FemX-1 tumour around blood vessels.

**Figure 8:**
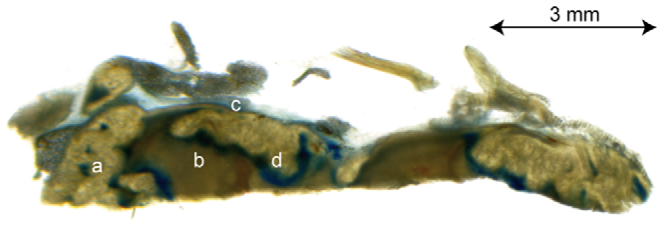
Evans Blue-Albumin complex distribution in vibratome sections of FemX-1 melanoma. Evans Blue-Albumin complex positive areas (blue) are recognizable at blood vessels in vital tumour tissue (a), at the tumour margin (c) and the transition (d) between vital tumour tissue and necrosis (b).

To study the mechanism of T84.1 penetration of the tumour tissue we investigated the tumour vessel permeability with an alternative technique. We injected the albumin-binding dye Evans Blue i.v. in FemX-1 melanoma bearing mice and investigated the dye distribution in vibratome sections of the tumour. Evans Blue-Albumin complex was present only around blood vessels, at the margin of the tumour, and in the transition zone between vital tumour tissue and central necrosis and thus was similar to the T84.1 binding (figure 8).

## Discussion

Identifying malignant tumours is one of the most important aims of molecular imaging. In order to bring molecular imaging techniques into clinical application, the technical approach has to be validated in suitable animal models first [30]. We therefore systematically investigated the CEACAM expression of twelve human cancer cell lines and their primary tumour xenografts in immunodeficient mice for their suitability to detect the tumour using an anti CEACAM antibody as a pre-requisite for imaging studies.

As human cancer cell lines often serve as the first choice for detecting human specific antigens, we analyzed CEACAM mRNA expression in a panel of human malignant cell lines. This was followed by the detection of their CEACAM protein expression in Western Blots. We found that pancreatic and colon (except of SW480) cancer cell lines have the highest expression levels of CEACAM with good correlation between mRNA amount and protein level in Western Blots. Similarly high protein levels were detected in melanoma cells, but their mRNA level was generally lower than that observed in the pancreatic and colon cancer cell lines. This discrepancy between mRNA and protein expression levels has already been observed e.g. in *Saccharomyces cerevisae* for the PUP2, and by mammals for circadian Period2 gene [31], [32]. RNA stability and/or translational efficiency between the cancer cell lines and different CEACAM family members could be a reason for the finding, that low mRNA level were associated with high protein levels.

A further discrepancy in CEACAM expression was observed between *in vitro* and *in vivo* CEACAM expression (figure 4). Except melanomas, all malignant cell lines showed a downregulation of CEACAM expression *in vivo* as compared to *in vitro*. CEACAM family members are mainly expressed by epithelial and endothelial cells at the free surface of their apical pole [33], [34]. In contrast to the three dimensional growth *in vivo* where tumour cells form a 3D conglomerate of cells with only a few free surfaces, almost all cultured cells have exposed free surfaces and therefore almost all cells display an apical cell pole enabling them to express CEACAM at this interface to the cell culture medium. This different growth behaviour results in a down regulation of CEACAM in the primary tumours compared to the cell culture growth.

The observation that the melanoma cell line showed the highest CEACAM expression level might not be so unexpected, as periodic acid-Schiff (PAS) reaction positive loops and networks indicating micro vascular channels within melanoma have been described [35]. As these microvascular channels are lined by the melanoma cells themselves and are not covered by endothelial cells, this apical surface might provide additional space for CEACAM expression. A further reason for the difference in expression can be found in tumour - stroma interactions. Cancer cells in a tumour xenograft are exposed to mouse extracellular matrix and mouse cells like blood vessel endothelia, lymphocytes and macrophages and also to mouse hormones. All these tumor-stroma interactions can potentially alter the expression of genes in the human tumour cell in xenografts [36]. The few regions of higher CEACAM expression *in vivo* in the xenograft of cell line T47D could be explained by the fact, that hypoxia could upregulate CEACAM expression via HIF1α signaling. A HIFα response element is located in the promoter region of CEACAM [37].

Another factor to be investigated is the accessibility of the monoclonal antibody to the CEACAM binding sites after i.v. application of the T84.1 antibody. Using labelled lectins as a probe, we have previously shown that vast differences in lectin binding site accessibility existed between the binding of the lectins to tissue sections and to the same tissue after i.v. injection of the same lectins [38]. We therefore investigated the presence of CEACAM binding sites *in vivo* using the CEACAM specific T84.1 mAB on FEMX-1 cells after its i.v. application. FEMX-1 cells were chosen as they readily grow in SCID mice and additionally show robust CEACAM expression *in vitro* and *in vivo* (figure 4). We could indeed show that the i.v. applied antibody reaches the target, but to a moderate extent only as its presence was limited to the direct vicinity of blood vessels (figure 5 and 7). Furthermore, melanoma is a particularly well suited malignancy to be investigated as CEACAM expression is positively correlated with metastasis formation [9]. Therefore more malignant cells express CEACAM-1 in melanoma, while in other tumour entities such as breast or prostate cancer CEACAM-1 expression is down-regulated during malignant progression [39], [40].

A known phenomenon of tumour xenografts is the uneven distribution of blood vessels within different areas of the tumour [41]. In our FemX-1 melanoma model the vascular density was more intense in the periphery than in the centre of the tumour xenograft (figure 8). The access of the T84.1 antibodies was limited to areas around blood vessels, resembling Kroghs' cylinder which shows the limits of oxygen diffusion in tissues [42]. Even the Evans Blue-Albumin complex with a lower mass weight of 67 kDa could only be found in the same area, as the injected T84.1 antibody (figure 8). All transport processes beyond this cylinder are not based on diffusion but on convection. Convection, however, is severely altered in tumours because of the absence of functional lymphatic vessels within the tumour. This leads to an accumulation of interstitial fluid within tumours resulting in a high interstitial fluid pressure of tumours which could be the pathophysiological mechanism behind this finding of a limited access of the antibody to the melanoma cells, despite the presence of microvascular channels in melanomas [43], [44].

The process of diffusion allows only the penetration of small molecules into the tumour, the size of the whole antibody molecule is obviously far too big to target cells behind the endothelial barrier as the target molecule CEACAM is clearly expressed in the tumour as shown in tissue sections. Therefore, the access of i.v. administrated anti CEACAM antibodies is limited to tumour cells surrounding blood vessels despite the fact, that many more tumour cells express CEACAM if studied in tissue sections. This lack of penetration might also explain why studies on the usage of anti CEA antibodies have not found acceptance in routine clinical practice [16], [17]. In order to target a wider range of CEACAM positive tumour cells, smaller target-specific molecules like DARPINs or nanobodies against CEACAM will be tested to see if they could reach the *in vivo* CEACAM binding sites to a greater extent. However, we could show that the target CEACAM could be studied with our melanoma FEMX-1 model and used for molecular imaging.

## References

[1] Edlund M, Gaardsvoll H, Bock E, Obrink B (1993). Different isoforms and stock-specific variants of the cell adhesion molecule C-CAM (cell-CAM 105) in rat liver.. Eur J Biochem.

[2] Kuespert K, Pils S, Hauck CR (2006). CEACAMs: their role in physiology and pathophysiology.. Curr Opin Cell Biol.

[3] Singer BB, Scheffrahn I, Obrink B (2000). The tumor growth-inhibiting cell adhesion molecule CEACAM1 (C-CAM) is differently expressed in proliferating and quiescent epithelial cells and regulates cell proliferation.. Cancer Res.

[4] Boulton IC, Gray-Owen SD (2002). Neisserial binding to CEACAM1 arrests the activation and proliferation of CD4+ T lymphocytes.. Nat Immunol.

[5] Schmitter T, Agerer F, Peterson L, Munzner P, Hauck CR (2004). Granulocyte CEACAM3 is a phagocytic receptor of the innate immune system that mediates recognition and elimination of human-specific pathogens.. J Exp Med.

[6] Kirshner J, Chen CJ, Liu P, Huang J, Shively JE (2003). CEACAM1-4S, a cell-cell adhesion molecule, mediates apoptosis and reverts mammary carcinoma cells to a normal morphogenic phenotype in a 3D culture.. Proc Natl Acad Sci U S A.

[7] Jantscheff P, Terracciano L, Lowy A, Glatz-Krieger K, Grunert F (2003). Expression of CEACAM6 in resectable colorectal cancer: a factor of independent prognostic significance.. J Clin Oncol.

[8] Jaques G, Bepler G, Holle R, Wolf M, Hannich T (1988). Prognostic value of pretreatment carcinoembryonic antigen, neuron-specific enolase, and creatine kinase-BB levels in sera of patients with small cell lung cancer.. Cancer.

[9] Thies A, Moll I, Berger J, Wagener C, Brummer J (2002). CEACAM1 expression in cutaneous malignant melanoma predicts the development of metastatic disease.. J Clin Oncol.

[10] Laack E, Nikbakht H, Peters A, Kugler C, Jasiewicz Y (2002). Expression of CEACAM1 in adenocarcinoma of the lung: a factor of independent prognostic significance.. J Clin Oncol.

[11] Steward AM, Nixon D, Zamcheck N, Aisenberg A (1974). Carcinoembryonic antigen in breast cancer patients: serum levels and disease progress.. Cancer.

[12] O'Brien CA, Pollett A, Gallinger S, Dick JE (2007). A human colon cancer cell capable of initiating tumour growth in immunodeficient mice.. Nature.

[13] Park JG, Frucht H, LaRocca RV, Bliss DP, Kurita Y (1990). Characteristics of cell lines established from human gastric carcinoma.. Cancer Res.

[14] Planque C, Kulasingam V, Smith CR, Reckamp K, Goodglick L (2009). Identification of five candidate lung cancer biomarkers by proteomics analysis of conditioned media of four lung cancer cell lines.. Mol Cell Proteomics.

[15] Wanebo HJ, Rao B, Pinsky CM, Hoffman RG, Stearns M (1978). Preoperative carcinoembryonic antigen level as a prognostic indicator in colorectal cancer.. N Engl J Med.

[16] Sharkey RM, Goldenberg DM, Goldenberg H, Lee RE, Ballance C (1990). Murine monoclonal antibodies against carcinoembryonic antigen: immunological, pharmacokinetic, and targeting properties in humans.. Cancer Res.

[17] Buraggi GL, Gasparini M, Seregni E (1991). Immunoscintigraphy of colorectal carcinoma with an anti-CEA monoclonal antibody: a critical review.. Int J Rad Appl Instrum B.

[18] Weissleder R (2002). Scaling down imaging: molecular mapping of cancer in mice.. Nat Rev Cancer.

[19] Lucka L, Fernando M, Grunow D, Kannicht C, Horst AK (2005). Identification of Lewis x structures of the cell adhesion molecule CEACAM1 from human granulocytes.. Glycobiology.

[20] Horoszewicz JS, Leong SS, Kawinski E, Karr JP, Rosenthal H (1983). LNCaP model of human prostatic carcinoma.. Cancer Res.

[21] Kaighn ME, Narayan KS, Ohnuki Y, Lechner JF, Jones LW (1979). Establishment and characterization of a human prostatic carcinoma cell line (PC-3).. Invest Urol.

[22] Schumacher U, Adam E (1997). Lectin histochemical HPA-binding pattern of human breast and colon cancers is associated with metastases formation in severe combined immunodeficient mice.. Histochem J.

[23] Carey TE, Takahashi T, Resnick LA, Oettgen HF, Old LJ (1976). Cell surface antigens of human malignant melanoma: mixed hemadsorption assays for humoral immunity to cultured autologous melanoma cells.. Proc Natl Acad Sci U S A.

[24] Fodstad O, Kjonniksen I, Aamdal S, Nesland JM, Boyd MR (1988). Extrapulmonary, tissue-specific metastasis formation in nude mice injected with FEMX-I human melanoma cells.. Cancer Res.

[25] Kohler S, Ullrich S, Richter U, Schumacher U (2010). E-/P-selectins and colon carcinoma metastasis: first in vivo evidence for their crucial role in a clinically relevant model of spontaneous metastasis formation in the lung.. Br J Cancer.

[26] Lange A, Gustke H, Glassmeier G, Heine M, Zangemeister-Wittke U (2011). Neuronal differentiation by indomethacin and IBMX inhibits proliferation of small cell lung cancer cells in vitro.. Lung Cancer.

[27] Kalinina T, Gungor C, Thieltges S, Moller-Krull M, Penas EM (2010). Establishment and characterization of a new human pancreatic adenocarcinoma cell line with high metastatic potential to the lung.. BMC Cancer.

[28] Smith PK, Krohn RI, Hermanson GT, Mallia AK, Gartner FH (1985). Measurement of protein using bicinchoninic acid.. Anal Biochem.

[29] Workman P, Aboagye EO, Balkwill F, Balmain A, Bruder G (2010). Guidelines for the welfare and use of animals in cancer research.. Br J Cancer.

[30] Marcus CD, Ladam-Marcus V, Cucu C, Bouche O, Lucas L (2009). Imaging techniques to evaluate the response to treatment in oncology: current standards and perspectives.. Crit Rev Oncol Hematol.

[31] Lehmann A, Janek K, Braun B, Kloetzel PM, Enenkel C (2002). 20 S proteasomes are imported as precursor complexes into the nucleus of yeast.. J Mol Biol.

[32] Lee C, Etchegaray JP, Cagampang FR, Loudon AS, Reppert SM (2001). Posttranslational mechanisms regulate the mammalian circadian clock.. Cell.

[33] Ergun S, Kilik N, Ziegeler G, Hansen A, Nollau P (2000). CEA-related cell adhesion molecule 1: a potent angiogenic factor and a major effector of vascular endothelial growth factor.. Mol Cell.

[34] Marquez RT, Baggerly KA, Patterson AP, Liu J, Broaddus R (2005). Patterns of gene expression in different histotypes of epithelial ovarian cancer correlate with those in normal fallopian tube, endometrium, and colon.. Clin Cancer Res.

[35] Thies A, Mangold U, Moll I, Schumacher U (2001). PAS-positive loops and networks as a prognostic indicator in cutaneous malignant melanoma.. J Pathol.

[36] Tlsty TD, Coussens LM (2006). Tumor stroma and regulation of cancer development.. Annu Rev Pathol.

[37] Kokkonen N, Ulibarri IF, Kauppila A, Luosujarvi H, Rivinoja A (2007). Hypoxia upregulates carcinoembryonic antigen expression in cancer cells.. Int J Cancer.

[38] Schumacher U, Borisch B, Welsch U, Lectins, Kocourek JFD (1990). Lectin binding in vivo versus lectin histochemistry..

[39] Riethdorf L, Lisboa BW, Henkel U, Naumann M, Wagener C (1997). Differential expression of CD66a (BGP), a cell adhesion molecule of the carcinoembryonic antigen family, in benign, premalignant, and malignant lesions of the human mammary gland.. J Histochem Cytochem.

[40] Luo W, Tapolsky M, Earley K, Wood CG, Wilson DR (1999). Tumor-suppressive activity of CD66a in prostate cancer.. Cancer Gene Ther.

[41] Carmeliet P, Jain RK (2000). Angiogenesis in cancer and other diseases.. Nature.

[42] Kreuzer F (1982). Oxygen supply to tissues: the Krogh model and its assumptions.. Experientia.

[43] Heldin CH, Rubin K, Pietras K, Ostman A (2004). High interstitial fluid pressure - an obstacle in cancer therapy.. Nat Rev Cancer.

[44] Jain RK, Baxter LT (1988). Mechanisms of heterogeneous distribution of monoclonal antibodies and other macromolecules in tumors: significance of elevated interstitial pressure.. Cancer Res.

